# Age and Growth of *Phoxinus grumi* Berg, an Endemic Fish in the Turpan Basin

**DOI:** 10.3390/ani16121806

**Published:** 2026-06-11

**Authors:** Xiaoqiang Li, Minghui Gao, Zhiyuan Luo, Xin Wang, Wei Guo

**Affiliations:** 1 Xinjiang Key Laboratory for Ecological Adaptation and Evolution of Extreme Environment Organisms, College of Life Sciences, Xinjiang Agricultural University, Urumqi 830052, China; 13523574346@163.com (X.L.); gmhxxzj@xjau.edu.cn (M.G.); 18146913651@163.com (X.W.); 2 Xinjiang Shuifa Ecology Technology Co., Ltd., Urumqi 831000, China; 18167802598@163.com

**Keywords:** *P. grumi* Berg, Turpan Basin, sex ratio, age, length–weight relationship, von Bertalanffy

## Abstract

*P. grumi* Berg, an endemic species of the Turpan Basin, constitutes an important component of the local aquatic biodiversity. This study aimed to identify the growth model that best describes the growth characteristics of *P. grumi* Berg and provide baseline biological information on this poorly studied species in Turpan. The research was conducted in the Turpan Basin in November 2024 and March 2025. A total of 527 specimens were collected, with body lengths ranging from 39.0 to 96.0 mm and body weights ranging from 0.69 to 16.00 g. The female-to-male sex ratio was 3.6:1. Age determination based on otolith analysis indicated that individuals ranged from 1 to 5 years old, with age groups 2–3 years predominating in the population, with age groups 2–3 years predominating in the population. The length–weight relationship for females was W = 4.0674 × 10^−5^L^2.7695^ (R^2^ = 0.8028), for males was W = 1.2645 × 10^−5^L^3.02^ (R^2^ = 0.9103), and for the combined population was W = 1.9513 × 10^−5^L^2.9321^ (R^2^ = 0.8221). The von Bertalanffy growth parameters were estimated as follows: L_∞_ = 102.25 mm, k = 0.331, t_0_ = −0.7548, Φ = 2.3297, t_i_ = 3.5392; L_∞_ = 105.82 mm, k = 0.2157, t_0_ = −0.6737, Φ = 4.4503, t_i_ = 3.3829. The results suggest that the *P. grumi* Berg population may currently exhibit an increasing population trend; however, the age structure was dominated by younger individuals, indicating a relatively unstable population structure. These findings enrich the basic biological information available for *P. grumi* Berg and provide an important scientific basis for future conservation and management strategies.

## Simple Summary

The Turpan Basin in western China is a desert region where water is scarce and the environment is fragile. Here lives a tiny fish, *Phoxinus grumi* Berg, which is found nowhere else on Earth. Despite being a protected species in Xinjiang since 2022, very little is known about its basic biology. To help protect this fish, we studied 527 individuals collected in late 2024 and early 2025. By examining their ear stones (otoliths)—which record age like tree rings—we determined their growth patterns. We found that most fish in the population are young (2–3 years old), with very few older individuals. While the population currently seems to be growing, this age structure is unstable and makes the species vulnerable to environmental changes, such as reduced water flow from glaciers due to climate change. This study provides the first detailed look at the growth of this little-known species. Our findings offer essential information for conservationists to help safeguard this unique fish and its fragile desert oasis home in the Turpan Basin.

## 1. Introduction

The Turpan Basin is a relatively enclosed inland basin located in the extremely arid region of northwestern China [1]. Characterized by a continental desert climate and surrounded by high mountain ranges, the basin reaches a maximum elevation of 5445 m above sea level, whereas its lowest point lies 154.31 m below sea level [2,3]. The Turpan watershed is among the driest and most water-insecure areas in China [3]. Its rivers and water sources primarily derive from snowmelt in the mountains, which flows through surface streams and the Karez system [4]. Fish diversity within this watershed is low, with only four native species recorded: *B. microphthalma*, *T. yarkandensis*, *T. minuta*, and *P. grumi* Berg [5]. Owing to its desert climate and strong dependence on glacial and snowmelt water, the Turpan watershed is particularly vulnerable to climate change. Future warming is expected to be more pronounced in high-latitude and high-mountain regions than the global average, potentially intensifying hydrological instability and ecological vulnerability in arid freshwater ecosystems [4,6,7,8,9]. This vulnerability is further exacerbated by ongoing land degradation, desertification, and increasing water demands associated with agricultural irrigation [10]. Consequently, existing lakes and oases face threats to their ecological balance [11,12]. Under these severe environmental conditions, the risk of declining fish populations in the region continues to rise [13]. Among the native fish species, *P. grumi* Berg appears to be particularly vulnerable to these environmental changes.

**Figure 1:**
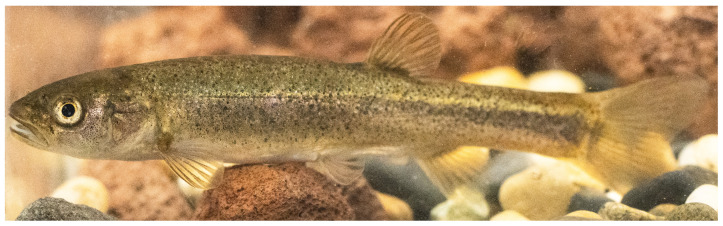
*P. grumi* Berg.

*P. grumi* Berg (Figure 1) belongs to the order Cypriniformes, family Cyprinidae, subfamily Leuciscinae, and genus *Phoxinus*. It is a small oxyphilic cold-water fish species with a maximum body length of 11.7 cm. The body length is 4.15–5.41 times the body height and 3.61–4.80 times the head length. The dorsal surface is gray-brown with numerous small black spots, whereas the ventral surface is white. A dark longitudinal stripe extends along the dorsal midline and the lateral side of the body, which is also marked by larger purple spots [5,14]. Due to its distinctive characteristics and limited habitat, this species is endemic to the Turpan Basin, occurring exclusively in oasis regions such as Dacaohu and Grape Valley [5]. In 2022, it was designated as a Class II Protected Animal in the Key Protected Wildlife Inventory of the Xinjiang Uygur Autonomous Region. The species was first described by the Soviet scholar Berg in 1907 and later documented by Li Sizhong et al. in 1966 [14]. No specimens were collected between 1967 and 2012 [5] until Xie Peng successfully gathered samples in 2019 [15].

The study of fish age and growth is fundamental to the fields of fish biology and ecology [16]. Accurate determination of fish age is a critical prerequisite for evaluating population dynamics and population structure [17,18]. Age data provide the basis for estimating key demographic parameters, including growth rate, mortality, and recruitment. Growth performance is crucial for fish survival and serves as a significant indicator for evaluating the abundance of aquatic resources [19]. Furthermore, age and growth information can assist fishery managers in evaluating the effectiveness of catch quotas and other fishery management strategies [20].

Ongoing research has resulted in the development of an increasing number of direct and indirect methods for estimating fish age and growth. However, identifying the most appropriate method remains challenging, as different techniques may yield inconsistent results when applied to the same population [21]. Among these methods, otoliths are generally considered the most reliable structures for age determination because they are widely believed to preserve the complete growth history of an individual [22,23]. Compared with scales and vertebrae, otoliths are metabolically inert and are less susceptible to resorption, regeneration, or environmental influences, thereby providing more stable and accurate annuli for age determination [23,24]. In small-bodied cyprinid fishes such as *P. grumi* Berg, scales often exhibit indistinct annuli due to slow growth and crowding of circuli in older individuals, while vertebral annuli may be difficult to interpret consistently. By contrast, otoliths generally provide clearer and more reliable growth increments, improving the precision and consistency of age estimation [25,26]. Currently, the von Bertalanffy growth model [27] is the most commonly utilized framework [28,29]. This model, grounded in bioenergetics, seeks to describe how catabolism and anabolism influence growth throughout a fish’s life cycle from a biological perspective [27,30]. Analyzing the age structure and growth rates of fish can unveil critical characteristics, such as life history and population structure, which are essential for assessing population health and maintaining ecosystem stability and biodiversity [31,32,33]. Investigating the age and growth of fish enhances the understanding of their resource status and establishes a foundation for conducting fishery resource assessments [34].

This study conducted sampling surveys of the *P. grumi* Berg population in the Turpan Basin from 2024 to 2025. Age was determined through otolith annuli analysis, and body lengths at age were back-calculated from otolith radius measurements. The von Bertalanffy growth parameters were then estimated using back-calculated length-at-age data to characterize the growth pattern of the species. The objective of this research was to comprehensively investigate the individual biological traits of this species, thereby addressing the existing knowledge gap and elucidating its age and growth patterns.

## 2. Materials and Methods

### 2.1. Sampling

**Figure 2:**
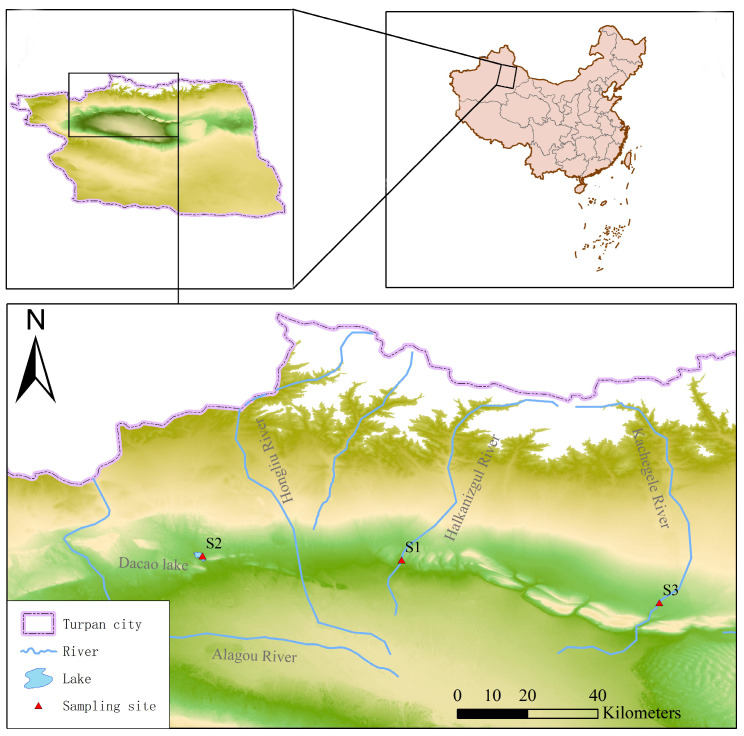
The sampling areas for *P. grumi* Berg in Turpan were S1 (Grape Valley), S2 (Dacaohu), and S3 (Lianmuqin Bazaar Village).

Sampling occurred in November 2024 and March 2025. To ensure comparability among sites and seasons, all surveys were performed at the same sampling sites using identical fishing gear and standardized sampling duration throughout the study period. A variety of fishing gear, including drift gillnets (mesh size: 2 cm), customized gillnets (mesh size: 2 cm), and minnow traps (mesh size: 2 cm), facilitated the collection of 527 specimens of *P. grumi* Berg from sites within the Turpan Basin. These sites included Grape Valley (89.2501° E, 43.0134° N), Dacaohu (88.7424° E, 43.0116° N), and Lianmuqin Bazaar Village (89.9123° E, 42.8901° N), as illustrated in Figure 2.

The total body length (*L*, mm) and body weight (*W*, g) of all specimens were measured on-site. Length measurements were taken with an accuracy of 1.0 mm, while weight measurements were recorded with an accuracy of 0.1 g. Specimens were fixed and preserved in a 10% formaldehyde solution and subsequently transported to the laboratory for immediate processing. In this species, gonadal development is not entirely synchronized among individuals of similar size, and some individuals remain at immature or resting stages despite having comparable body lengths. During these stages, gonadal characteristics are often poorly differentiated, which hinders reliable sex determination. In addition, because part of the sampling was conducted near the breeding season, post-spawning gonadal regression further reduced the distinguishability of sexual characteristics in some individuals. Therefore, specimens with insufficiently developed or ambiguous gonadal characteristics were conservatively classified as “undetermined sex”. Microotoliths served as the materials for age determination. Of the 527 collected specimens, otoliths were successfully extracted from 116 individuals for further analysis.

### 2.2. Otoliths

**Figure 3:**
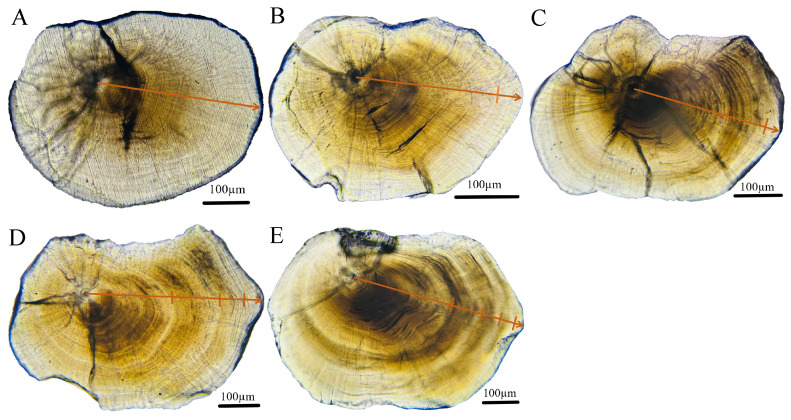
Measurement orientation of otoliths in *P. grumi* Berg, where images (**A**, **B**, **C**, **D** and **E**) correspond to otoliths of age groups 0^+^, 1^+^, 2^+^, 3^+^ and 4^+^, respectively. The arrows indicate the longitudinal axis of the otolith, and the lines mark the boundary between the light and dark bands.

The micro-otoliths were embedded in GTS polyester resin (manufactured by Woss Chemical Company, Morbi, India, styrene content 35–40%) mixed with MEKP hardener, mounted on glass slides with their convex side facing upward. After drying for 12 h, the otoliths were ground using waterproof abrasive papers with grit sizes ranging from 1500 to 3000 (Kafuwell (Hangzhou) Industrial Co., Ltd., Hangzhou, China). This grinding process necessitated frequent observation and adjustment under a microscope until the otolith core was reached [35,36]. Once the growth center became distinctly visible, images were captured using a digital camera (MicroPublisher 5.0 Real-Time Viewing, Qimaging, Surrey, BC, Canada) attached to an Olympus BX53 microscope (Olympus, Tokyo, Japan), and the otolith radius was measured, with the measurement direction indicated in Figure 3. Ages were determined by examining the transition between translucent and opaque zones, which are assumed to be deposited annually. These annuli were designated as 0^+^, 1^+^, 2^+^, and so forth. For age-group analysis, these designations (0^+^, 1^+^, 2^+^, …) were statistically treated as ages 1, 2, 3, and so on, respectively. In addition, samples with damaged or severely blurred otoliths were classified as unsuccessful age readings. Age-reading precision was evaluated using the Coefficient of Variation (CV) method recommended by Campana (2001) [24]. Two readers independently performed blind readings based on unified criteria. Individual and mean CV values were then calculated from the paired readings.

### 2.3. Relationship Between Radius of Auricle and Body Length

The relationship between body length (*L*, mm) and otolith radius (*OR*, μm) was evaluated using five models: linear, power, exponential, logarithmic, and quadratic. The optimal regression equation was determined by identifying the model with the highest coefficient of determination (*R*^2^). To assess differences in otolith morphometric characteristics between females and males, analysis of covariance (*ANCOVA*) was conducted with body length as the covariate [37]. The modified Lee equation was utilized to back-calculate body length at specific ages [38]. The reliability of the back-calculated length-at-age estimates was assessed by comparing observed and back-calculated length-at-age values. Differences between the two datasets were analyzed using paired-samples *t*-tests, with statistical significance defined as *p* < 0.05. This equation is expressed as follows:

(1) $$L_{i}\;=\;\frac{\alpha \;+\;\beta \;\times \;{OR}_{i}}{\alpha \;+\;\beta \;\times \;OR}\;\times \;L$$

*L* represents the measured body length (mm), *OR* denotes the otolith radius (μm), *L_i_* indicates the back-calculated body length at age *i* (mm), *OR_i_* refers to the radius corresponding to annulus *i* (μm), and *α* and *β* are the intercept and slope of the body length–otolith radius regression equation, respectively.

### 2.4. Relationship Between Length and Weight

Common growth equations were employed to examine the growth characteristics. A two-sample Kolmogorov–Smirnov test was performed to determine whether the body length and body weight distributions differed between female and male individuals [39,40]. Parameters a and b were estimated by fitting the log-transformed data through the least-squares method.

The power–law relationship between body length and weight is described by the following equation:

(2) $$W=aL^{b}$$

The parameters *a* and *b* were derived by fitting the log-transformed data using the least-squares method. An analysis of covariance (*ANCOVA*) was conducted to assess differences in the estimated slopes between female and male samples [37] (*p* < 0.05). Additionally, a *t*-test [41] was performed to evaluate whether the allometric coefficient b significantly deviated from the theoretical isometric value of *b* = 3. A significant difference indicated allometric growth, while a lack of difference suggested isometric growth.

The *t*-test formula is as follows:

(3) $$t\;=\frac{SD(L)}{SD(W)}\times \frac{\left(/b-3/\right)}{\sqrt{1-R^{2}}}\times \sqrt{n-2}$$

Here, *SD*(*L*) and *SD*(*W*) represent the standard deviations of the logarithm of body length and body weight, respectively; *n* is the sample size, and *R*^2^ is the coefficient of determination.

### 2.5. Growth Equation

The growth parameters were estimated using back-calculated body lengths at age derived from otolith annuli measurements. The Ford–Walford transformation was applied as a linearization procedure to obtain the initial estimates of the von Bertalanffy growth parameters based on age–length data. The von Bertalanffy growth model was subsequently fitted using linear least-squares regression. Its equation is:

(4) $$L_{t}=L_{\infty }[1-e^{-k(t-t_{0})}]$$

(5) $$W_{t}={bW}_{\infty }[1-e^{-k(t-t_{0})}{]}^{b}$$

To further predict growth trends, the first and second derivatives of the growth equation were calculated, resulting in the growth rate equation, the growth acceleration equation, and the equation for the inflection point of growth [41,42]. The growth rate equation is:

(6) $$\frac{dL}{dt}\;={\;L}_{\infty }ke^{-k(t-t_{0})}$$

(7) $$\frac{dW}{dt}={bW}_{\infty }ke^{-k(t-t_{0})}[1-e^{-k(t-t_{0})}{]}^{b-1}$$

The growth acceleration equation is:

(8) $$\frac{d^{2}L}{dt^{2}}={-L}_{\infty }k^{2}e^{-k(t-t_{0})}$$

(9) $$\frac{d^{2}W}{dt^{2}}={bW}_{\infty }k^{2}e^{-k(t-t_{0})}[1-e^{-k(t-t_{0})}{]}^{b-2}[be^{-k(t-t_{0})}-1]$$

The inflection point age of growth was estimated based on the body weight growth equation derived from the von Bertalanffy growth model. The inflection point corresponds to the age at which the growth rate of body weight reaches its maximum. It was calculated according to the following equation:

(10) $$t_{i}\;=\frac{lnb}{k}+t_{0}$$

In the equation, *L_t_* represents the body length of an individual at age *t*, while *Wt* denotes the body weight of the individual at the same age. *L_∞_* indicates the asymptotic length, and *W_∞_* signifies the asymptotic weight. The parameter *t*_0_ refers to the theoretical age at which both length and weight are zero, representing a hypothetical age. The curvature parameter of the growth curve is denoted by *k*, and b represents the exponent of the length–weight relationship. The term *dL/dt* indicates the growth rate in body length, whereas *dW/dt* reflects the growth rate in body weight. Additionally, *d*^2^*L/dt*^2^ represents the acceleration in body length growth, and *d*^2^*W/dt*^2^ denotes the acceleration in body weight growth.

The growth performance index was calculated using the formula [43]:

(11) $$\Phi \;=\;logk\;+\;2logL_{\infty }$$

## 3. Results

### 3.1. Frequency Distributions of Length and Weight

**Figure 4:**
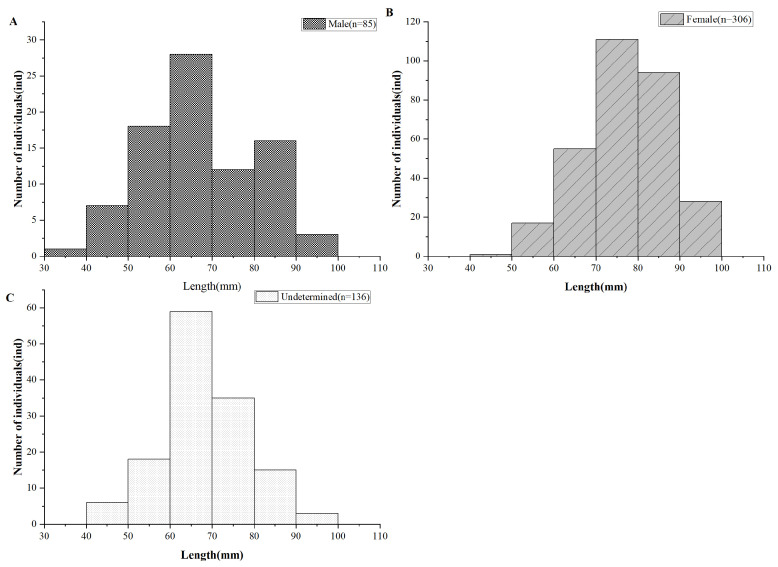
Distributions of the frequencies of L of *P. grumi* Berg, where (**A**) represents male population, (**B**) represents the female population, and (**C**) represents an undetermined gender group.

Among the 527 specimens of *P. grumi* Berg collected, 85 were male, 306 were female, and 136 were of undetermined sex, yielding a sex ratio of 3.6:1 (♀:♂). The frequency distribution of body length (*L*) is illustrated in Figure 4. The total length of the specimens ranged from 39.0 to 96.0 mm, with a mean length of 71.7 ± 11.4 mm. The predominant length class was 60.0–90.0 mm, which comprised 90.70% of the total samples. Males exhibited a length range of 39.0–95.0 mm, while females ranged from 48.0 to 96.0 mm. A significant difference in body length was detected between females and males (two-sample Kolmogorov–Smirnov test, *D* = 0.397, *p* < 0.001).

**Figure 5:**
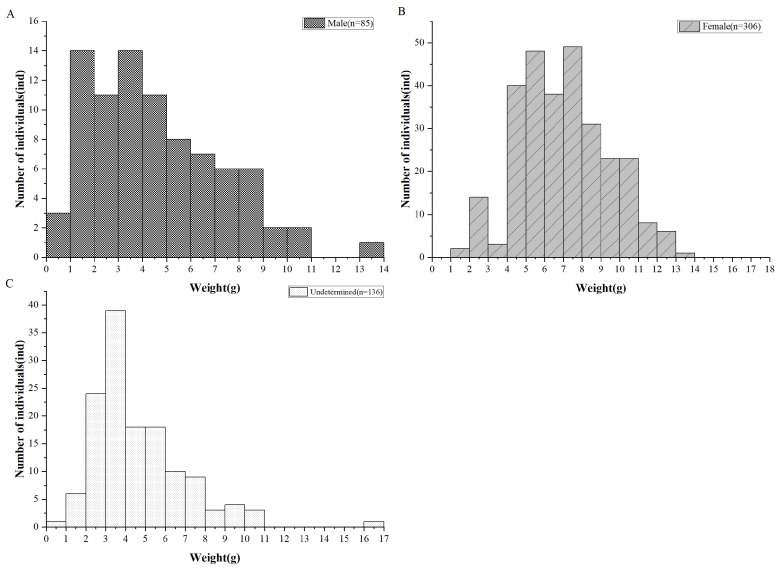
Distributions of the frequencies of W of *P. grumi* Berg, where (**A**) represents male population, (**B**) represents the female population, and (**C**) represents an undetermined gender group.

The frequency distribution of body weight (*W*) is illustrated in Figure 5. Sample weights varied from 0.69 to 16.00 g, with a mean weight of 5.81 ± 2.69 g. The predominant weight class was 2.00–9.00 g, which constituted 77.23% of the total samples. The weight range for females was 1.87–13.60 g, while for males it was 0.69–13.80 g. A significant difference in body weight between females and males was identified (two-sample Kolmogorov–Smirnov test, *D* = 0.388, *p* < 0.001).

### 3.2. Frequency Distributions of Age

**Figure 6:**
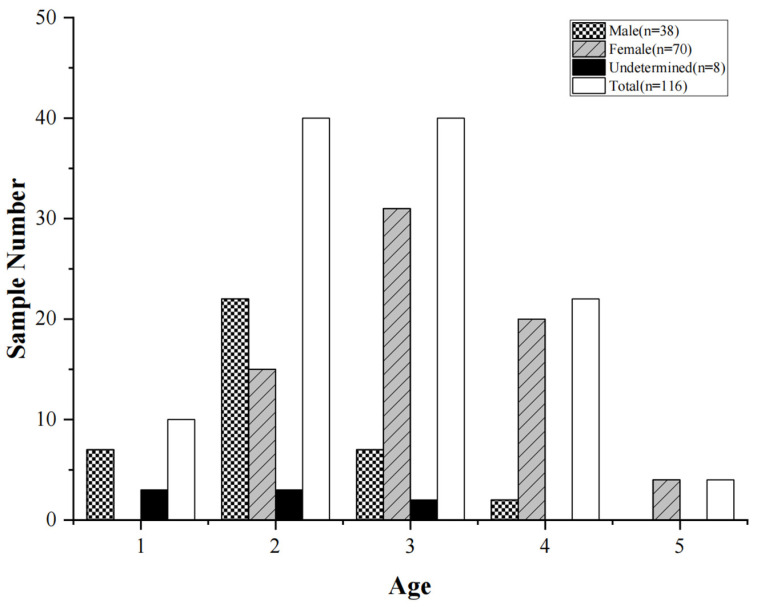
Distributions of the frequencies of Age of *P. grumi* Berg, where 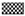 represents male population, 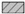 represents the female population, 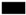 represents an undetermined gender group, and 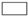 represents the whole group.

Age was determined via otolith examination for 137 of the 527 collected specimens. Among these, age was successfully undetermined for 116 individuals, while the determination was unsuccessful for the remaining 21 individuals (11 females and 10 males). The average coefficient of variation (CV) between the two readers was 5.4%, indicating high precision and consistency in age determination. The age composition of the *P. grumi* Berg population is illustrated in Figure 6. The youngest captured individual was 1 year old, and the oldest was 5 years old, with the predominant age class being 2–3 years. For males, the dominant age class was 1–3 years, whereas for females, it was 2–4 years. The mean age was 2.11 years for males and 3.19 years for females.

### 3.3. Length–Weight Relationship

**Figure 7:**
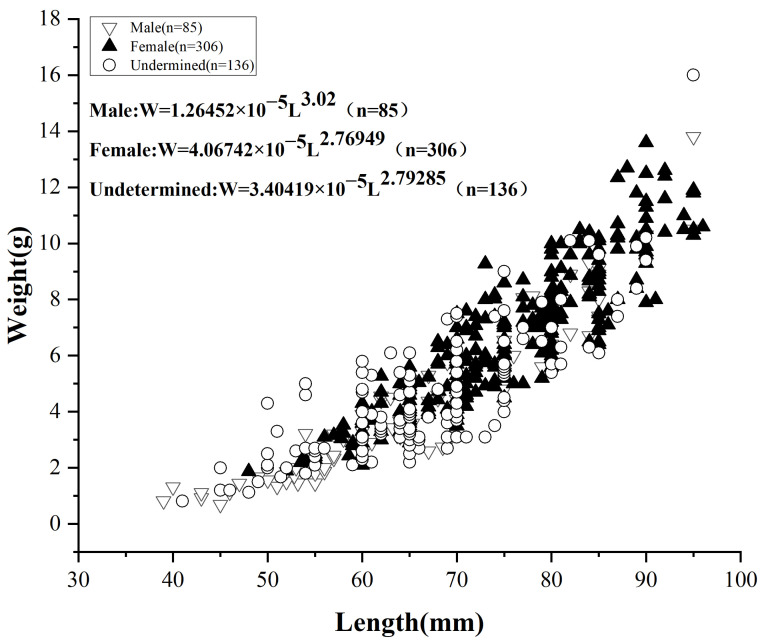
Length–weight relationship of male and female *P. grumi* Berg, where 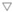 represents male population, 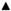 represents the female population, and 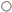 represents undetermined population.

For all 527 specimens, the power function relationship between body length (*L*) and body weight (*W*) is represented by the following equation (Figure 7):

Overall population:

(12) $$W=1.9513\times 10^{-5}L^{2.9321}(R^{2}=0.8221,\;n=527)$$

ANCOVA demonstrated a significant difference in the length–weight relationship between male and female *P. grumi* Berg (F = 10.827, *p* < 0.05). Males exhibited isometric growth, as the estimated b-value did not differ significantly from the theoretical value of 3 (t = 0.549, *p* > 0.05). Conversely, females showed negative allometric growth, with the estimated b-value being significantly lower than 3 (t = 3.577, *p* < 0.05). Here, allometric growth refers specifically to the proportional relationship between body length and body weight rather than age-related population growth patterns.

### 3.4. Relationship Between Otolith Radius and Age of Fish

**Figure 8:**
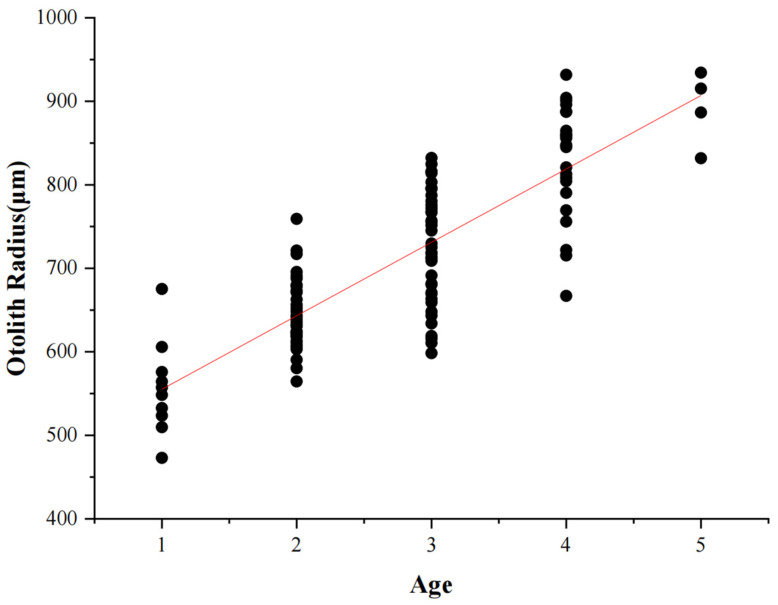
Relationships between otolith radius and age of fish. 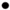 represents the samples, and the solid line indicates the fitted relationship.

The relationship between otolith radius along the major axis and age was examined (Figure 8). A linear model was fitted to the data, revealing a relationship between otolith radius and log-transformed age: *OR* = 87.9243*A* + 467.343 ($R^{2}=0.6902)$. These findings suggest that otolith radius increases with age.

### 3.5. Relationship Between Otolith Radius and Body Length

**Figure 9:**
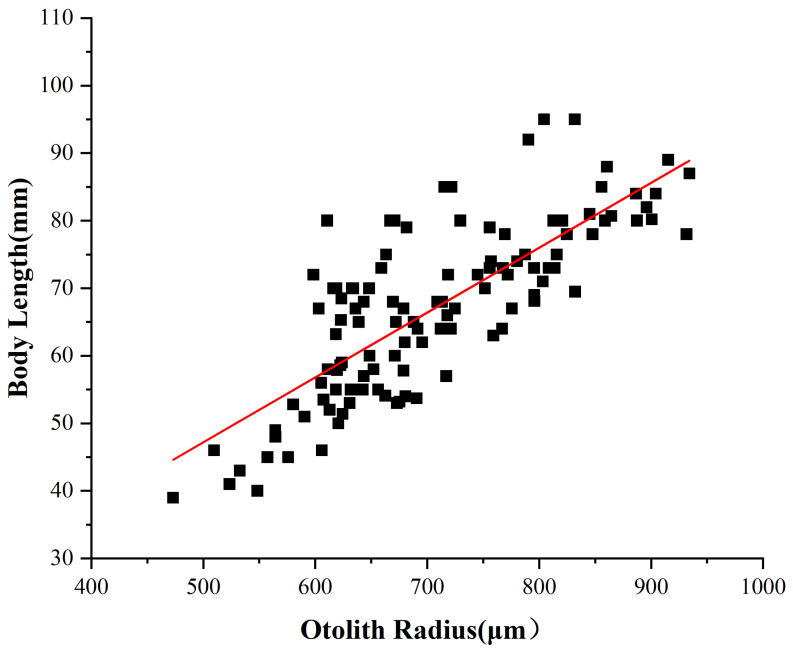
Relationships between otolith radius and body length of fish. 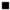 represents the samples, and the solid line indicates the fitted relationship.

In examining the relationship between body length (*L*) and otolith radius (*OR*), five regression equations were constructed using various mathematical models. Among these, the linear regression model demonstrated the highest coefficient of determination for fitting the relationship between *L* and *OR*. The resulting equation is expressed as *L* = 0.0096*OR* − 0.0784, $n=116,\;R^{2}=0.6164,\;p<0.001$ (Figure 9).

### 3.6. Back-Calculation of Growth

Analysis of covariance revealed no significant difference in otolith radius between females and males (*ANCOVA*, *F* = 1.167, *p* > 0.05); consequently, female and male data were pooled for further analysis. The total length and otolith radius measurements of the 116 *P. grumi* Berg specimens were incorporated into the following formula:

(13) $$L_{i\;}=\;\frac{0.0784\;+\;0.0096\;\times {\;OR}_{i}}{0.0784\;+\;0.0096\;\times \;OR}\;\times \;L$$

The back-calculated body lengths at each age are presented in Table 1. A paired *t*-test was conducted to compare the observed body lengths with the back-calculated length-at-age values. No significant difference was detected between the two datasets (*t* = −2.40, *df* = 2, *p* > 0.05), indicating that the back-calculated length-at-age values were generally consistent with the observed body lengths.

### 3.7. Growth Equation, Growth Rate, and Growth Acceleration Equation

Because significant differences were detected in the body length and body weight distributions between females and males, the two sexes were analyzed separately in this section (see Section 3.1 and Section 3.2 for the results of the significance tests). Using the back-calculated body length data for each age group of *P. grumi* Berg, linear equations were fitted relating *L_t_*_+1_ to *L_t_* and ln(*L_∞_* − *L_t_*) to t through the least-squares method. The following parameters were derived:

♂:

(14) $$L_{t+1}=20.529+0.806L_{t}(R^{2}=0.9336)$$

(15) $$ln(L_{\infty }-L_{t})=4.496-0.246t(R^{2}=0.9995)$$

♀:

(16) $$L_{t+1}=28.774+0.7186L_{t}(R^{2}=0.9994)$$

(17) $$ln(L_{\infty }-L_{t})=4.3776-0.331t(R^{2}=0.9999)$$

The following parameters can be derived. ♂: *L_∞_* = 105.82 mm, *k* = 0.2157, *t*_0_ = −0.6737; ♀: *L_∞_* = 102.25 mm, *k* = 0.331, *t*_0_ = −0.7548.

The growth performance index and the age at the inflection point for *P. grumi* Berg are detailed in Table 2.

Based on the established length–weight relationships—♂: W = 1.2646 × 10^−5^L^3.02^ and ♀: W = 3.9607 × 10^−5^L^2.7695^—the corresponding asymptotic weights were calculated as ♂: W_∞_ = 16.45 g and ♀: W_∞_ = 14.57 g. Consequently, the von Bertalanffy growth equations for *P. grumi* Berg ware derived as follows:

♂:

(18) $$L_{t}=105.82(1-e^{-0.2157(t+0.6737)})$$

(19) $$W_{t}=16.45(1-e^{-0.2157(t+0.6737)}{)}^{3.02}$$

♀:

(20) $$L_{t}=102.25(1-e^{-0.331(t+0.7548)})$$

(21) $$W_{t}=14.57(1-e^{-0.331(t+0.7548)}{)}^{2.7695}$$

The first and second derivatives of the growth equations for body length and weight with respect to time yielded the growth rate and acceleration equations for body length and weight, respectively:

♂:

(22) $$\frac{dL}{dt}=22.825e^{-0.2157(t+0.6737)}$$

(23) $$\frac{dW}{dt}=(10.716e^{-0.2157(t+0.6737)})[1-e^{-0.2157(t+0.6737)}{]}^{2.02}$$

(24) $$\frac{d^{2}L}{dt^{2}}=-4.923e^{-0.2157(t+0.6737)}$$

(25) $$\frac{d^{2}W}{dt^{2}}=(2.311e^{-0.2157(t+0.6737)})[1-e^{-0.2157(t+0.6737)}{]}^{1.02}[3.02\times e^{-0.2157(t+0.6737)}-1]$$

♀:

(26) $$\frac{dL}{dt}=33.845e^{-0.331(t+0.7548)}$$

(27) $$\frac{dW}{dt}=(13.387e^{-331(t+0.7548)})[1-e^{-0.331(t+0.7548)}{]}^{1.7695}$$

(28) $$\frac{d^{2}L}{dt^{2}}=-11.203e^{-0.331(t+0.7548)}$$

(29) $$\frac{d^{2}W}{dt^{2}}=(4.431e^{-0.331(t+0.7548)})[1-e^{-0.331(t+0.7548)}{]}^{0.7695}[2.7759\times e^{-0.331(t+0.7548)}-1]$$

**Figure 10:**
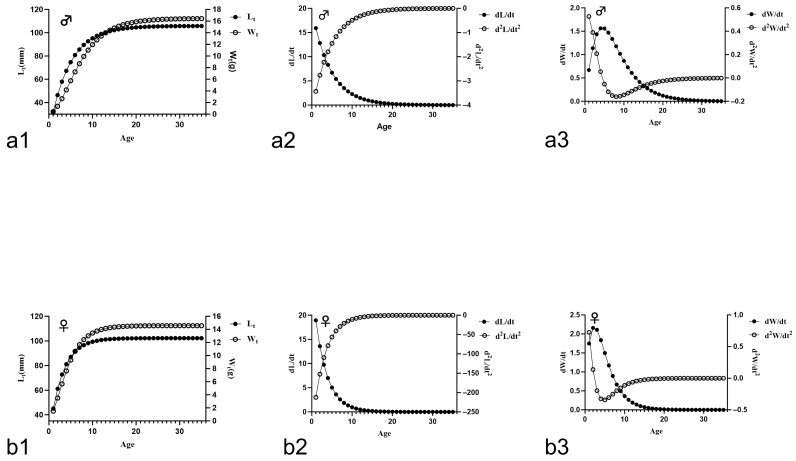
The von Bertalanffy growth curve, along with the growth rate and acceleration of *P. grumi* Berg, is illustrated. The parameters (**a**) and (**b**) denote male and female, respectively. Curve (**1**) represents the von Bertalanffy growth curve, Curve (**2**) depicts the growth rate, and Curve (**3**) illustrates the growth acceleration.

Figure 10 illustrates the von Bertalanffy growth curve, as well as the growth rate and growth acceleration for body length (*L*) and body weight (*W*) of *P. grumi* Berg.

## 4. Discussion

This study systematically investigated the age and growth characteristics of *P. grumi* Berg for the first time, providing essential baseline data for understanding the life-history traits and population structure of this species.

### 4.1. Population Structure

This study demonstrated that the body length distribution and age composition of *P. grumi* Berg comprised individuals ranging from 1 to 5 years of age, with the 2–3 year age group predominating and accounting for 68.3% of the sampled population. These results are consistent with previous studies on *P. phoxinus* ujmonensis, *P. percnurus*, *P. lagowskii*, and *P. oxycephalus* [15,44,45]. Among the 527 specimens examined, body lengths ranged from 39 to 96 mm, with 425 individuals (80.6%) concentrated within the 60–90 mm size class. Notably, both the maximum (96 mm) and minimum (39 mm) body lengths recorded in the present study were markedly smaller than those previously reported for this species [5,14]. The asymptotic lengths (*L_∞_*) derived from model fitting were 102.25 mm for males and 105.82 mm for females in this study, which are lower than the maximum body length reported for this species in the mid-20th century [14]. However, these differences should be interpreted with caution, as they may reflect differences in sampling methods, sample size, or sampling locations among studies. Additional long-term monitoring data and comparisons with historical population size distributions are required before drawing conclusions regarding temporal changes in body size.

The growth rate analysis demonstrated that *P. grumi* Berg exhibited relatively rapid growth during the juvenile stage, followed by a gradual decline with increasing age. Furthermore, the growth rate of younger females was significantly higher than that of males. Rapid early growth is generally regarded as an adaptive strategy that enhances juvenile survival and ecological competitiveness during vulnerable early developmental stages, particularly in cold-water or environmentally constrained habitats [46,47]. In contrast, the decline in growth rate during later life stages may reflect a progressive shift in energy allocation from somatic growth toward maintenance metabolism and reproductive development, which is commonly observed in fishes undergoing life-history transitions [48].

The growth acceleration curve showed an initial increase followed by a gradual decrease after reaching its peak, indicating that the efficiency of somatic growth declined with age. The occurrence of peak growth acceleration may correspond to a critical life-history transition stage during which individuals allocate substantial energy to somatic growth before progressively shifting physiological investment toward maturation-related processes, a pattern commonly associated with biphasic growth in fishes [49]. Similar growth dynamics and ontogenetic transitions have also been reported in cyprinid fishes, in which inflection points and changes in allometric growth are considered indicators of developmental and ecological adaptation [50].

Although the observed body size reduction and population structure may suggest potential environmental pressure or ecological changes affecting the population, caution is required when interpreting long-term population trends. Because the present study was based primarily on age and growth characteristics from a limited sampling period, additional long-term monitoring and reproductive investigations are necessary to further evaluate the population dynamics and ecological status of *P. grumi* Berg.

### 4.2. Growth Characteristics

The *b* value reflects the relationship between body length and body weight. This parameter varies not only among different species but also among populations of the same species and across different developmental stages. Such variation is commonly associated with differences in stomach fullness, feeding conditions, and gonadal development status [51]. In the present study, the *b* value was estimated at 3.02 for male *P. grumi* Berg and 2.7695 for females, indicating evident sexual differences in growth patterns. Males exhibited isometric growth, whereas females showed negative allometric growth.

The growth coefficient *k* serves as a key indicator of population growth rate. Branstetter (1987) [52] categorized growth coefficients into three classifications: 0.05–0.1 for slow-growing species, 0.1–0.2 for moderately growing species, and 0.2–0.5 for fast-growing species. The *k* values for male and female *P. grumi* Berg were 0.2157 and 0.331, respectively, indicating that this population can be classified as fast-growing. Notably, the *k* value for males surpassed the previously reported range for the genus *Phoxinus* (0.101–0.251), while the *k* value for females fell within the medium-to-high range of this spectrum [15,44]. The asymptotic length *L_∞_* for males (105.82 mm) exceeded that for females (102.25 mm), although the corresponding *k* value was relatively lower. Consequently, males require a longer duration to attain their asymptotic length compared to females. This observation indirectly implies that females prioritize energy allocation for fecundity and reproductive success, resulting in a more rapid somatic growth rate [53,54,55]. In contrast, males may allocate more resources to growth and competition, contributing to a comparatively slower growth rate [54,56]. Beyond habitat factors, the size range of captured specimens can also affect *k* estimation. Therefore, adjusting fishing gear and selecting suitable sampling sites are essential for obtaining accurate parameter estimates [24].

The growth performance index (*Φ*) serves as a tool for comparing growth performance among species within the same genus [57,58]. In this study, the growth performance indices (*Φ*) for female and male *P. grumi* Berg were found to be 2.3297 and 4.4503, respectively. The index for females is significantly lower than the previously reported range for the genus *Phoxinus* (3.47–4.09), whereas the index for males exceeds this range [14,44]. Consequently, males within the *P. grumi* Berg population demonstrated superior growth performance compared to females. However, because the sample sizes differed between males and females, the potential influence of sampling imbalance on growth parameter estimation cannot be completely excluded. Therefore, the sex-specific differences in *Φ* values should be interpreted with caution. In addition, the growth performance index is highly sensitive to the estimation of von Bertalanffy growth parameters, particularly *L_∞_* and *K*, and relatively small variations in these parameters may substantially affect *Φ* values [59,60]. Thus, the unusually high *Φ* value observed in males may partly reflect uncertainty in parameter estimation and should be interpreted conservatively.

The inflection point age serves as a reliable framework for understanding the transition from early to late life stages in fish. Research indicates that the inflection point age in fish correlates closely with the age at sexual maturity and the onset of senescence, while also being influenced by factors such as fluctuations in water temperature and food availability [61,62]. Mathematical calculations determined the inflection point ages for female and male *P. grumi* Berg to be 3.5392 and 3.3829 years, respectively. Among the catch, 36 male individuals (94.74%) were below their inflection point age of 3.3829 years, while 46 female individuals (65.71%) were below their inflection point age of 3.5392 years. Both proportions exceeded 60%. Additionally, the mean ages of the male and female *P. grumi* Berg populations (♂: 2.11 years; ♀: 3.19 years) were lower than their respective inflection point ages, suggesting a current trend toward a younger age structure within the *P. grumi* Berg population.

The sex ratio serves as a critical indicator of the population structure in fish. By influencing the reproductive potential and spawning behavior of the breeding population, it indirectly impacts population dynamics [63]. Typically, an increase in the proportion of males within a fish population can result in a decline in reproductive potential and efficiency [64]. In the *P. grumi* Berg population examined in this study, the female-to-male sex ratio was 3.6:1. This ratio exceeds the previously reported female-to-male sex ratios for *P. oxycephalus* (1:2.08), *P. lagowskii* (1:1.33), and *P. percnurus* (1:2.08) [44,45]. Consequently, it can be inferred that the current *P. grumi* Berg population exhibits relatively high reproductive potential and efficiency. This may reflect an ecological adaptation aimed at maintaining population stability amidst harsh and variable habitat conditions, as well as various anthropogenic disturbances [62]. However, sex determination was unsuccessful for 136 specimens, which may have affected the representativeness and accuracy of the estimated sex ratio. Therefore, the observed female-to-male ratio should be interpreted cautiously.

Although 527 otolith samples were collected in this study, only 116 were successfully used for age determination because many otoliths were damaged during preparation or exhibited severely blurred annuli that prevented reliable interpretation. Although age-reading precision was evaluated using independent blind readings and the coefficient of variation (CV) method, the relatively low success rate may still have introduced potential sampling bias and affected the representativeness of the estimated age structure and growth parameters. Therefore, the present results should be interpreted with caution. Future studies employing improved otolith preparation techniques and larger effective sample sizes are needed to further validate the age structure and growth characteristics of *P. grumi* Berg.

## 5. Conclusions

The investigation into the age determination and growth characteristics of *P. grumi* Berg revealed that the current population demonstrates a relatively rapid growth rate and a high female-to-male ratio, which may indicate relatively strong reproductive capacity and active population recruitment. However, the population structure is predominantly composed of younger individuals, suggesting a potentially unstable age distribution. These findings not only enhance the fundamental biological data available for *P. grumi* Berg but provide valuable reference information for future conservation and management efforts.

## Figures and Tables

**Table 1:** Measured body length and back-calculated body length at each age class of *P. grumi* Berg.

Age Class	Sample Number	Average of Measured Body Length (mm)	Back-Calculated Body Length (mm)			
			L1	L2	L3	L4
2	40	58.65	43.73			
3	40	71.09	44.10	58.07		
4	22	81.38	45.64	60.83	72.21	
5	4	88.75	46.22	62.19	72.84	81.58
Weighted mean (mm)			44.92	60.37	72.53	81.58

**Table 2:** *P. grumi* Berg growth performance index and growth inflection age.

Sex	*Φ*	*t_i_*
Male	4.4503	3.3829
Female	2.3297	3.5392

## Data Availability

Data will be made available on request.
